# Medical Applications of Diode Lasers: Pulsed versus Continuous Wave (cw) Regime

**DOI:** 10.3390/mi12060710

**Published:** 2021-06-17

**Authors:** Michał Michalik, Jacek Szymańczyk, Michał Stajnke, Tomasz Ochrymiuk, Adam Cenian

**Affiliations:** 1 Centrum Medyczne MML, 00-112 Warszawa, Poland; michalim@mml.com.pl; 2 Department of Dermatology, Medical University of Warsaw, 82a Koszykowa Street, 02-008 Warsaw, Poland; szymanczyk.jacek@gmail.com; 3 Department of Physical Aspects of Ecoenergy, The Szewalski Institute of Fluid-Flow Machinery, Polish Academy of Sciences, 14 Fiszera Street, 80-952 Gdansk, Poland; michal.stajnke@imp.gda.pl (M.S.); tomasz.ochrymiuk@imp.gda.pl (T.O.)

**Keywords:** laser diodes, pulsed and continuous wave (cw) regimes, medical applications, dermatology, laryngology

## Abstract

The paper deals with the medical application of diode-lasers. A short review of medical therapies is presented, taking into account the wavelength applied, continuous wave (cw) or pulsed regimes, and their therapeutic effects. Special attention was paid to the laryngological application of a pulsed diode laser with wavelength 810 nm, and dermatologic applications of a 975 nm laser working at cw and pulsed mode. The efficacy of the laser procedures and a comparison of the pulsed and cw regimes is presented and discussed.

## 1. Introduction

We are approaching the 60th anniversary of laser medical applications. Shortly after the invention ruby lasers (with wavelength 694.3 nm) in the 1960s, Goldman et al. [1] started using it as therapy for melanoma, a human skin disease [2]. Later, in the 1980s, more powerful lasers, such as CO_2_ lasers, argon lasers, and Nd:YAG lasers, were applied in the field of surgery (including laparoscopic), ophthalmology, dermatology, oncology, etc. An important step forward was the implementation of selective photothermolysis in dermatology by Anderson and Parrish [3], which are based on pigment-specific, short-pulsed lasers, e.g., Q-switched lasers.

Diode lasers (DLs), which first appeared in 1962, are still the most energy efficient and cost effective lasers. Therefore, they have found more and more applications in the field of medical therapies. Initially, DLs were not so popular as they gave power only in the order of mW. Diode lasers were used mainly for photobiomodulation (PBM)—previously also known as biostimulation or low-level laser therapy, LLLT— procedures, as well as for photodynamic therapy, where the wavelength is more crucial than high power [4,5]. Although PBM therapy was implemented by Endre Mester et al. [6] in 1967, for several decades it was mistrusted by many medical laser specialists. Only recently, after recognition of the role of cytochrome c oxidase in the mitochondrial respiratory chain as a primary chromophore and the introduction of the concept of “retrograde mitochondrial signaling”, have attitudes changed. The significance of PBM in cell culture studies, resistance to fungal infections, mitigation of the side-effects of cancer therapy, pain and inflammation therapies, wound healing, muscle performance, etc. has become clearer. For example, Kowalec et al. [7] studied the Ceralas D15 diode laser which delivers optical power at 980 nm for wound and ulcer healing applications. The treatment enhanced wound healing and improved patient satisfaction and wellbeing. The study [8] confirmed the photomodulation efficacy of low power DL radiation at 740 nm (previously proven to be effective in wound healing) for therapy of dry eye disease. The radiation can improve corneal surface, alleviate inflammation through decreasing of the neutrophils levels, etc. Espey et al. [9] demonstrated that PBM using 665 nm pulsed (200 ns) radiation from a DL with fluence 4 and 6 J/cm^2^ results in a significant increase in sperm motility and velocity within 120 min post-irradiation.

High power (above 5 W) DL applications for surgery have taken place in dermatology (see, e.g., [10]) and oral surgical procedures (e.g., [11]). Bass [10] demonstrated DLs ability to eliminate vascular lesions, in a therapy called photosclerosis or thermocoagulation, using a DL emitting 810 nm wavelength radiation and has achieved satisfactory effects without scarring. The applied laser fluence during the square wave pulse (5–15 ms) was 14–42 J/cm^2^. The pulse interval was 32 ms (~31 Hz). Lesions treated included telangiectasias, spider veins, capillary dermal malformation and a cutaneous venous malformation. Telangiectasias were most responsive, usually disappearing after one treatment. Later, similar effects were achieved using a 980 nm diode laser by Desiate et al. [12]. Saetti et al. [13], after performing 22 endoscopic DL (810 nm) treatments of congenital subglottic hemangiomas, concluded that it is the safest and most effective (95% efficacy) therapy. The same cw DL radiation was used by Ferri et al. [14] to successfully treat Tis and T1 glottic carcinomas; all patients were able to eat without aspiration, as soon as the second day. Mittnacht et al. [15] applied DL radiation with power up to 450 W and λ = 808 and 940 nm to lung tissue. The laser with wavelengths 810 nm (3.5 W cw, 200 µm fiber) was used to treat oral Pyogenic granuloma [16].

More recently, Lee et al. [17] studied the efficacy of laser tonsillectomy using a 1940-nm laser working with a fluence of 12 W. The mean time for the procedure was 22.6 min and a notable reduction in pain at one week postoperative was elicited. Kang et al. [18] applied DL radiation with wavelength 1940 nm for the treatment of nasal congestion due to hypertrophied nasal turbinates. As the absorption coefficient of 1940-nm radiation in tissue is very high, the laser ablates tissue more precisely with less thermal damage. This clinical feasibility trial included eight patients with inferior turbinate hypertrophy. A rather low laser power of 4.5 W was applied leading to good medical results. In order to increase cutting efficiency of 940 nm DL, Agrawal et al. [19] studied the effect of various external chromophores (beetroot extract, erythrosine dye, hibiscus extract) applied on animal tissues. Staining of tissues with 3% erythrosine dye improved the efficacy of a 940 nm diode laser, by introducing sharper, wider cuts and clean incision with minimal charring when compared to beetroot, hibiscus, and saline chromophore.

In addition, the efficacy of 532-nm DL was investigated by treating a 50-year-old Korean female with oral erythro-leucoplakia [20]. Two months after the DL treatment, using a power of 6 W and 25 ms pulse, the operated region was well-healed without any significant scar contracture. Diode lasers emitting at wavelength 808 nm and different fluencies (12–14 J/cm^2^) were tested for hair removal efficacy [21]; 30 ms laser pulses at a fixed rate of 7 Hz were applied. No significant difference was observed for both applied fluencies including patient comfort. The treatments were tolerated well without anaesthesia. The feasibility of a diode laser emitting at 1470 nm for blood vessel sealing was studied by Im et al. [22]. It was found that a power of 20 W and irradiation time of 5–10 s are adequate for effective sealing of blood vessels, although the higher power is required to cut the vessels.

Diode lasers with a central wavelength in the range 980 ± 10 nm have not been widely used up until now in high power clinical therapies. Romanos et al. [11] examined the wound healing after the application of a diode laser (980 nm) in oral surgical procedures, such as removal of soft tissue tumors, frenectomies, excision of gingival hyperplasias, vestibuloplasties, hemangioma removal, and periimplant soft tissue surgery. Laser radiation was applied both in pulsed and cw regime, with and without contact to the tissue. The advantages of this procedure were good coagulation properties; lack of bleeding, pain, scar tissue formation or swelling; and good wound healing. A few other examples related to otolaryngology procedures are known: turbinate reduction, nasal polypectomy, ablation of an oral papilloma, and photocoagulation of nasal telangiectasias [23]. Schmedt et al. [24] has studied endovenous laser treatment of saphenous veins using a diode-laser emitting light of wavelength 980 nm which was transported via a 600 μm bare tipped optical fibre. Telangiectasias were most responsive, usually disappearing after one treatment [12]. Reynaud et al. [25] applied the 980 nm laser in laser-assisted lipolysis and Weiss et al. [26] in laser-assisted liposuction. Tunçel et al. [27] used DL (4–9 W) cw radiation to treat early glottic cancer and a year later Karasu et al. [28] applied DL radiation (3–5 W cw) to vocal fold polyps.

A Ceralas D15 diode laser delivering up to 15 watts of optical power at 980 nm using a quartz fiber delivery system was used to treat benign laryngeal lesions at office-based (outpatient) surgery—see [29]. Laser radiation (at power 12 W superpulse mode) was applied to a lesion through the working channel (3.7 mm in diameter) of the video fiberoptic esophagoscope. Some treated lesions such as: vocal polyps, leucoplakia, laryn- geal hair showed significant improvement, yet required repeated procedures. On the other hand, patients with contact granuloma, subglottic stenosis and tracheal lesions showed partial remission with laser surgery. Recently, Karkos et al. [30,31] demonstrated the efficacy of a new ‘‘Π’’ surgical technique (using 980 nm DL laser, 4–9 W) postoperatively to improve quality of voice and swallowing. It was proven that the 980 nm diode laser appears to be safe and ‘‘friendly’’. Excellent long-term decannulation rates together with no significant deterioration in voice quality was achieved. Prażmo et al. [32] confirmed a positive effect of the repeated 980 nm laser pulsed irradiation (100 Hz) on intracanal *Enterococcus faecalis* biofilm elimination.

The effects of 975 nm radiation of dermatologic DL (in pulsed and cw mode) developed in IMP PAN was studied using optical phantoms of skin [33] before its clinical application [34]. Further research comparing the interaction effects of radiation at 532, 975, and 1064 nm was performed and reported by Milanic et al. [35]. It was concluded that the risks of the epidermis or subcutaneous tissue overheating are significantly reduced.

The aim of this paper is to describe and analyse the medical application of diode lasers operating in pulsed and cw regimes, with a special focus on laryngological or dermatological therapies. The results related to the authors’ experience in the field are presented and discussed, including first simulations of dermatologic treatment. The advantage of pulsed laser application is discussed and its limitations are analysed.

## 2. Materials & Methods

The medical therapies analysed here were performed for several hundred patients treated in a private clinic, the Medical Centre MML in Warsaw (in the field of laryngology) and the private dermatology practice of Dr J. Szymańczyk, in cooperation with the Institute of Fluid-Flow Machinery PAS in Gdańsk.

The Institute developed a dermatologic diode laser emitting at 975 nm, working at cw or pulsed regime—pulse lengths 100 ns−300 ms, and laser output power 20 W [36], which was later implemented for therapies of neurofibroma and hemangiomas [34]. The second diode laser applied in MML Centre generated radiation with wavelength 810 nm and a pulse duration 4 s. In both cases, high efficacy of laser treatments was registered. Efficacy of procedure was defined as the ratio of the number of patients with positive effects of treatment therapy to the total number of procedures performed.

Besides medical treatments, the theoretical modelling and analyses of laser radiation interaction with neurofibroma blisters were performed. Therefore, the classic fluid-solid interaction problem is simulated and solved, in which the use of the monolithic method [37] is justified. First of all, non-trivial coupling of the thermal-FSI type [38] is considered, with the laser beam providing a heat stream to the tumor surface. There is an unstable flow of heat stream through various types of tissues to the tumor interior filled with fluid. This fluid heats up and there is a phase change, combined with a rapid increase in pressure, which results in a significant non-linear increase in tumor volume due to the hyperelastic properties of the skin. The tumor eventually explodes after some time, less than the time it takes to reach the pain threshold. The Arbitrary Lagrangian-Eulerian (ALE) description gives a proper foundation for monolithic methods in which simultaneous solution for all unknowns of the coupled fluid/solid system [39] and all interaction effects between the dependent equations are included. The set of balance equations in the well-known ALE form [40,41] are solved

(1) $$\frac{\partial }{\partial t}\left\{\begin{array}{c} \rho \\ \rho v\\ \rho e \end{array}\right\}+\mathrm{div}\left\{\begin{array}{c} \rho v\\ \rho v⊗v\\ \rho ev \end{array}\right\}=\mathrm{div}\left\{\begin{array}{c} 0\\ t\\ tv+q \end{array}\right\}+\left\{\begin{array}{c} 0\\ \rho b\\ \rho bv \end{array}\right\}\;,$$

where ρ is the density of the continuum particle, v  is velocity of the continuum particle, $e=c_{v}T+\frac{1}{2}v^{2}$ is total energy, $c_{v}$ is specific heat at constant volume, T is temperature of the continuum particle, t is the Cauchy stress flux, q=λ·T∇ is the molecular heat flux defined by Fourier law (λ is thermal conductivity coefficient), and b is the earth acceleration. The Cauchy stress flux can be divided into an elastic part and a diffusive part:

(2) $$t=P+\tau^{c}\;,\;$$

where P is an elastic momentum flux which is reversible and $\tau^{c}$ is a total diffusive momentum flux which describes irreversible phenomena. Below the first introductory results of simulations are presented and analyzed.

## 3. Results

Here, the results of diode laser treatments performed in MML Centre (laryngology) and a private dermatology practise are presented and discussed.

### 3.1. Pulsed Diode Laser 810 nm (5 W Fluence and Pulse Duration 4 s) in Laryngology Applications in MML Centre

**Figure 1:**
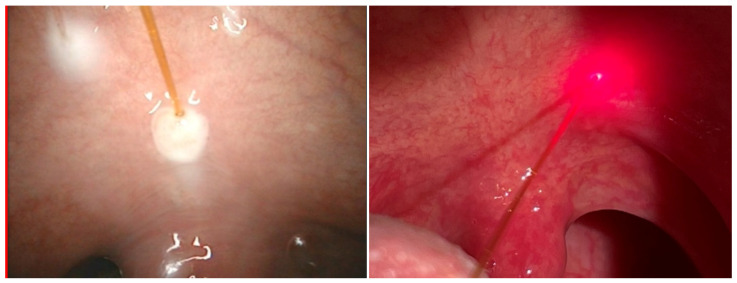
Introduction of laser fiber into a soft palate.

(i) Laser-assisted somnoplasty using the palisade technique, a method of treatment for snoring and sleep apnea, is characterized by high efficacy, a short recovery period, and minimal risk of complications [42]. The method is implemented for palatoplasty, surgery of palatoglossal and palatopharyngeal arch, and uvuloplasty. During the procedure, the diode laser fibre is introduced into the soft palate (see Figure 1), which results in the formation of linear intra-parenchymal adhesions that stiffen the palate and shift it in the vertical plane. The therapy results in the prevention of tissue vibration during sleep, which, in turn, leads to increased sleep comfort and maximally widened airways. There are several advantages for application of this laser-assisted procedure, e.g., it enables a shorter surgery time (30–40 min), under local anaesthetic conditions. Shortly after the procedure, the patient can be discharged.

**Figure 2:**
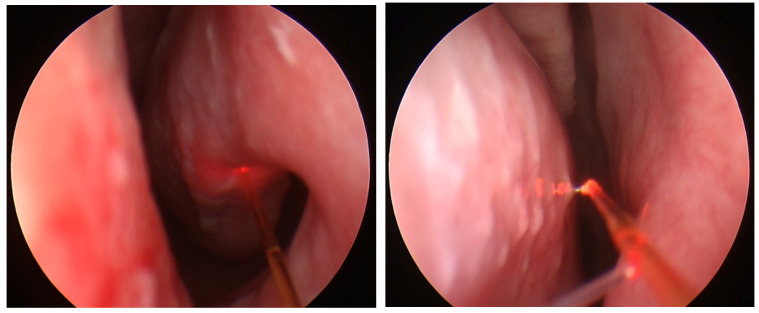
Laser assisted separation of adhesions in nasal septum.

**Figure 3:**
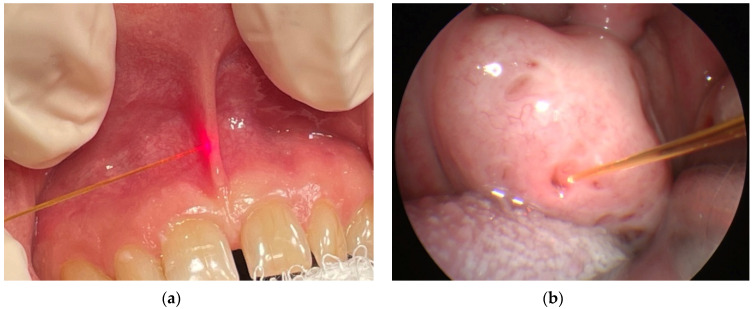
Frenectomy of labial frenulum (**a**) and laser-assisted closure of tonsillar crypts (**b**).

In years 2007–2020, 84 diode laser-assisted somnoplasty procedures using palisade technique were performed. Complete clinical response was observed in 77 cases, and a partial response was seen in seven cases. The efficacy of the therapy reached 92%.

(ii) Separation of adhesions in nasal septum is needed due to postoperative complications—see Figure 2. The adhesions being postoperative (iatrogenic) cicatrix appear between nasal conches and septum and inhibit normal air flow. After laser assisted separation, instead of the usual tamponade, a gel dressing, which dissolves after a certain period, is applied as sufficient. The laser procedure is safer for the patient and gives better results. From 2007 to 2020, 51 laser-assisted separation procedures were performed. Complete clinical response was observed in 49 cases, and a partial response was seen in two cases. The efficacy of the therapy reached 97%.

(iii) Laser assisted frenuloplasty, a surgery for a short frenulum and frenectomy of labial frenulum is a simple, sensitive and safe medical procedure (Figure 3a). It is preceded by a local anaesthesia. The diode laser assisted therapy is bloodless and painless due to the character of laser radiation tissue interaction (increased coagulation). During the period 2007–2020, 62 diode laser-assisted frenectomy procedures were performed. Complete clinical response was observed in 61 cases, and a partial response was seen in one case, giving a procedure efficacy of 98%.

(iv) Laser-assisted closure of tonsillar crypts after removal of debris (known as tonsil stones) resulting from bacterial and viral infections (see Figure 3b). After the debris removal a diode laser fiber is introduced, which enables shrinking and closing of crypts. This is an ambulatory (also known as office-based or Outpatient) procedure under local anaesthetic, and is painless and bloodless. During the period 2007–2020, 31 diode laser-assisted closures of tonsillar crypts were performed. Complete clinical response was observed in 29 cases, and a partial response was seen in two cases, giving an efficacy of 96%.

**Figure 4:**
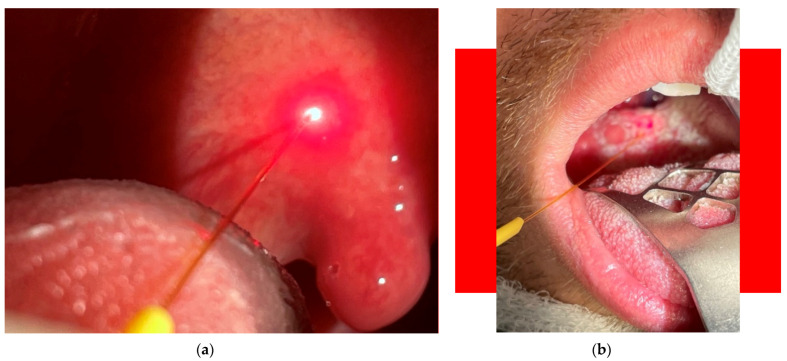
Laser-assisted (**a**) haemostasis and (**b**) surgery of laryngopharynx.

**Figure 5:**
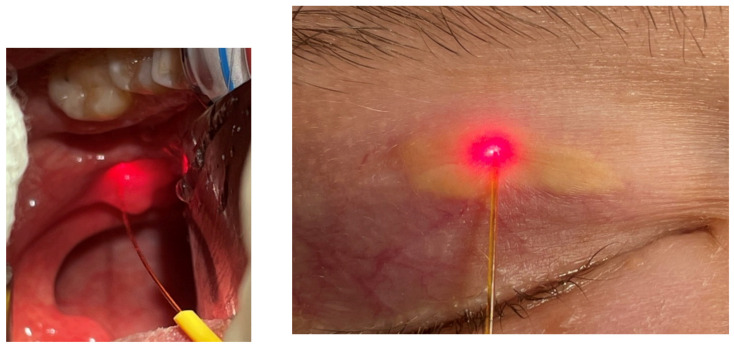
Laser-assisted removal of cancerous tissue.

**Figure 6:**
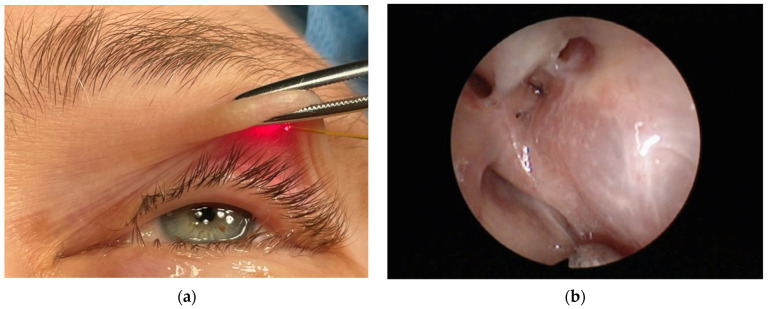
The laser-assisted (**a**) blepharoplasty and (**b**) nasolacrimal duct anastomosis 12 months after procedure.

(v) Laser-assisted haemostasis (coagulation) results from interaction of 810 nm radiation of diode laser with the blood and lymphatic vessels—see Figure 4a. The process enables bloodless procedures and eliminates haemorrhaging both during and postoperatively. The process efficacy reaches 100%.

(vi) Laser surgery of laryngopharynx and larynx (Figure 4b) enables sensitive and precise operation, removal of deteriorated tissues and protection of healthy ones. The separated tissue can be sent for histopathologic diagnostics. During the period 2007–2020, 54 laser surgery procedures were performed. Complete clinical response was observed in 50 cases, and a partial response was seen in 4 cases, resulting in an efficacy for the procedure of 93%.

(vii) Laser-assisted removal of cancerous changes/tissues (papilloma, polyps, haemangiomas, vocal nodules) enables precise operation and reaching narrow channels in nasal, sinus and other regions—see Figure 5. There is a low risk of thermal damage to tissue, so introduced wounds normally heal fast. The procedures are relatively fast and less invasive than standard ones. The laser haemostasis inhibits haemorrhage. During the period 2007–2020, 67 diode laser-assisted removals of cancerous changes were performed. Complete clinical response was observed in 64 cases, and a partial response was seen in three cases, giving an efficacy for the procedure of 95%.

(viii) The laser-assisted blepharoplasty (popular cosmetic eyelid surgery) is a me- dical/cosmetic procedure leading to correction of upper eyelid drooping (Figure 6a). It consists in removal of skin surplus from the upper eyelid. The procedure enables an increase of eyeshot (improved field of vision) and face rejuvenation. Its efficacy reaches 99%. During period 2007–2020, 97 diode laser-assisted blepharoplasty procedures were performed. Complete clinical response was observed in 96 cases, and a partial response was seen in one case.

(ix) Laser assisted dacryocystorhinostomy (DCR) was performed using a diode laser 810 nm, at power 8–10 W and pulses 0.5–1 s, in the case of patients with tear duct obstruction [43]. An elastic laser fiber 0.4 mm wide was introduced through the tear duct towards the lacrimal sac. The procedure was performed for 60 patients (44 women and 16 men) with average age 60.9 years. Positive effects were observed in the case of 96%, 75%, and 78%, after three, six, and 12 months, respectively (Figure 6b). In two cases the procedure was repeated and general efficacy increased to 81%. The intraoperative use of mitomycin C during the procedure of nasolacrimal duct anastomosis with diode laser increases its effectiveness [44].

Summing up, the utilisation of a 810 nm diode laser allows not only the removal of damaged tissue but it leads to haemostasis of blood vessels which in turn results in higher safety of therapies. This is of special importance when dealing with blood engorged tissues, where the risk of postoperative complications can be much higher.

### 3.2. Pulsed and Continuous (cw) Operation Regime of Diode Laser 975 nm Implemented for Therapy of Dermal Neurofibroma

**Figure 7:**
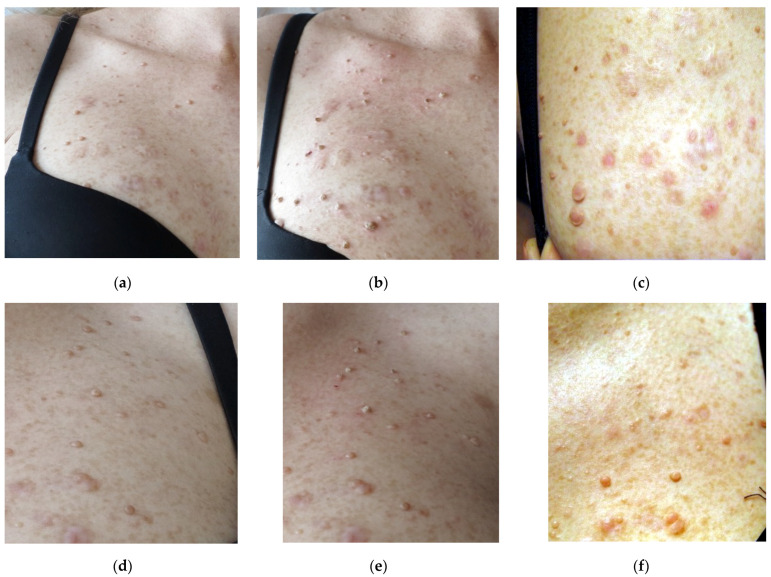
Laser therapy of dermal neurofibroma at right side of the décolleté area using DL radiation with wavelengths 975 nm: and cw power 10 W; (**a**) view before irradiation, (**b**) soon after irradiation (**c**) 7 weeks after laser treatment; and with pulsed power 15W (pulse 50 ms, 10 Hz) left side of the décolleté area (**d**) view before irradiation, (**e**) soon after irradiation (**f**) 7 weeks after laser treatment.

**Figure 8:**
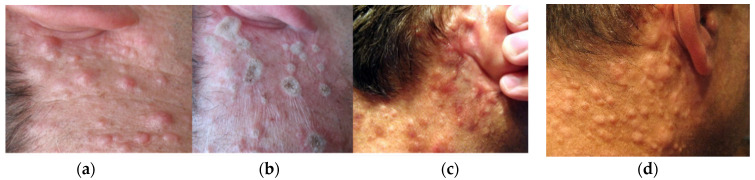
Laser therapy of dermal neurofibroma at right side of the neck below ear region using DL radiation with wavelengths 975 nm and continues wave, power: 12 W; (**a**) view before irradiation, (**b**) soon after irradiation (55 s), (**c**) 7 weeks after laser treatment, (**d**) one year after therapy.

In the case of patients affected by *dermal neurofibroma* disease, therapy proceeded at different levels of laser power in order to find the optimum conditions. Figure 7 presents the effects in the case, when laser radiation with cw power 10 W and 15 W pulsed regime (pulse 50 ms, 10 Hz) was applied to treat right side of the décolleté area. In the second case (see Figure 8), cw power 12 W was applied. The check after ~4–9 weeks have shown that the best therapeutic and cosmetic results have been achieved for cw power of 10 W. In the case of higher powers the healing period was longer as well as the cosmetic effect less desirable due to tendency to scarring. 

Application of lower radiation powers does not significantly improve the final therapeutic or cosmetic effect, i.e., by flattening of irradiated distortions or reduced tendency to scarring. It looks like the applied pulsed regime gives slightly better results (compare Figure 7c,f), the procedure is slightly less painful and better tolerated by patients. However, the procedure lasted longer. In the case of the patient presented in Figure 8, the effects one year after therapy may point to the need for therapy repetition.

Due to dolorability of the therapy using both diode (975 nm), Nd:YAG and Ho:YAG lasers the treatment was preceded by local anaesthesia with 1% of lignocaine. However, patients’ reactions and tolerance of these laser therapies were variable. In the case of diode laser (975 nm) patients do not experience real pain or any tissue warming despite deep coagulation. The tissue coagulation proceeds fast and effectively. The reaction to Ho:YAG laser irradiation (2100 nm) was different. Patients despite local anaesthesia very often suffered unpleasant high temperature effects due to the laser irradiation and coagulation process. The treatment (Ho:YAG laser irradiation) of the skin, necessary to achieve the required result, lasts significantly longer than in the case of the laser diode.

**Figure 9:**
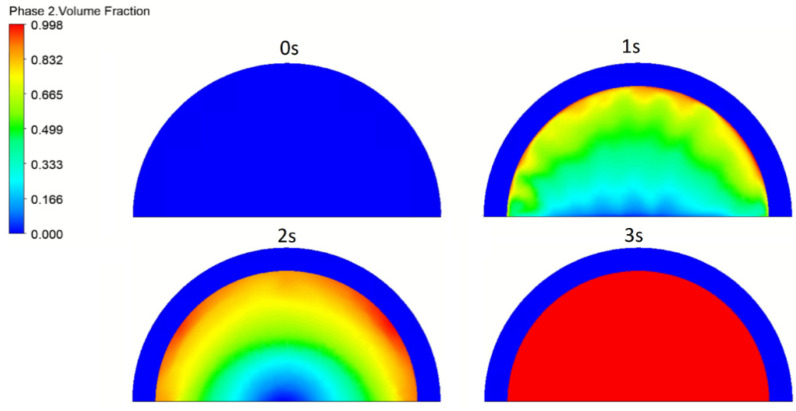
The simulated gas phase change during the period of 3 s.

In order to understand better the phenomena and mechanism of neurofibroma therapy, a theoretical modelling and analyses of laser radiation interaction with neurofibroma blister was performed. As mentioned in Section 2 the fluid contained in these cancerous blisters is heated by laser radiation and evaporates rapidly increasing pressure. The blister eventually explodes after some time, e.g., at least 3 s for blisters of 1.8 mm in diameter and more than 5 s for blisters 3 mm in diameter. These results correlate well with the results of introductory simulations based on the ALE model described above, e.g., the full evaporation of liquid in the neurofibroma blister occurred after 3 s of laser heating—see Figure 9.

**Figure 10:**
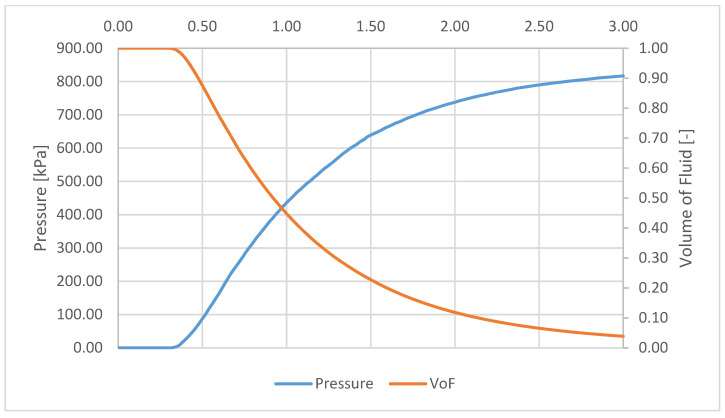
Relation between volume of fluid inside skin blister and pressure inside.

Figure 10 presents the relation between temporal evolution of pressure inside the cancerous blister and the volume of fluid which has not yet undergone phase change (evaporated). The maximum calculated pressure is 817 kPa, after 3 s of laser irradiation. At that moment, 96% of the liquid had already evaporated. At that moment, the pressure forces surpass the elastic forces and explosion results.

## 4. Discussions

Diode lasers with wavelengths of 810 ± 10 and 980 ± 10 nm are used in cw and pulsed modes. The radiation is transmitted to the operation field using optical fibre, which may or may not contact the tissue being treated. These DL’s promote less bleeding, cleaner and more adequate operative field, significant reduction in post-operative oedema associated with direct reduction in pain, and improvement in tissue repair (see, e.g., [45]). Besides, Hanke et al. [46] studied soft-tissue cutting-efficiency for DL emitting in the wavelengths (λ) range 400 to 1500 nm. They found that the cutting depth for 2.5 W laser radiation moving at the speed 2 mm/s is equal 530, 330, 260, 230 µ for λ = 445, 810, 980, 1064 nm, respectively. Total interaction zones change accordingly. The 980 nm radiation is slightly better absorbed by water than the 810 nm one, which results in a smaller interaction zone. For example, Goel et al. [47] stated “The diode laser 980 nm is usually preferred for DCR surgery as it provides a better ablation and narrower tissue area involvement versus 810 nm that creates better coagulation than the vaporization”. Table 1 presents various medical applications of the mentioned lasers.

Although in the paper we focus on diode laser application, in otolaryngology various lasers have been used, following the first (in the late 70s) implementation of an argon laser for inferior turbinate reduction. Lasers have been later successfully applied for a variety of nasal pathologies, such as epistaxis, inferior turbinate hypertrophy, nasal and paranasal tumors, skin lesions, and pathologies of the nasopharynx—see e.g., [53]. Although, Abiri et al. [54] pointed to the argon laser as the superior for some laryngology problems (caused, e.g., by hereditary haemorrhagic telangiectasia) other lasers such as Nd:YAG (second harmonic) and diode lasers also give good results. However, the application of CO_2_ laser radiation is limited due to the complexity of nasal anatomy and lack of appropriate elastic fibres.

The CO_2_, Nd:YAG (second harmonic), argon and diode lasers were also successfully applied to oral cavity and oropharyngeal lesions, such as hypertrophic gingivitis, chronic tonsillitis, benign and malignant tumors, etc. [55]. These lasers provide better haemostasis, greater cutting precision, and reduced postoperative edema when compared to other standard methods of surgery.

The first laser assisted dacryocystorhinostomy (DCR) was implemented (by Massaro et al. [56]) in order to increase the diameter of tear duct (nasolacrimal duct) whilst avoiding bleeding. The argon laser was used in order to generate a tear duct (4–6 mm wide), which allow tears from the lacrimal sac of the eye to reach the nasal cavity. Later, the advantage of various elastic fibres allowed the application of other wavelengths, e.g., 2120 nm of Ho:YAG laser [57], 810 [43] or 980 nm [58] diode lasers.

Fluence is a key parameter which should be carefully adjusted in order to cause minimal damage to tissues adjacent to the incision site. Another issue is related to pulse operation mode. It was observed during neurofibroma treatment that pulsed mode (50 ms, 10 Hz) was perceived by the patient as less painful than the cw regime. However, it led to a longer operation time. Besides, using a higher pulse power for a shorter period of time results in less tissue damage than using lower power for a longer period of time. This is of special importance for selective photothermolises studied by Anderson and Parrish [3], but the most popular diode laser used in medical therapies does not offer such possibilities. Therefore, cw mode operation is usually favoured in various therapies due to the reduced operation time. The exceptions are presented in Table 1.

## 5. Conclusions

The results of radiation tissue interactions depend upon the tissue absorption coefficient, the wavelength of the laser, power density, operation mode (including pulse lengths and frequency), and interaction time. Although these data are presently better described in various papers they are still not always fully provided.

From Table 1, it is clear that in the case of soft tissue surgery the cw operation mode is preferred by the medical staff. This is because of the limitations of pulse power in the most common diode lasers and its effect on operation time. However, one should remember that pulsed operation mode may result in less damage in tissue adjacent to the incision site. The 980 nm DL radiation may in some cases provide a better ablation and narrower tissue affected zone in relation to 810 nm laser which in turn will be better for coagulation.

Diode lasers are becoming increasingly popular in medical applications due to their small size, robustness and compactness, cost-effectiveness, and ease of operation as well as high efficiency (reaching up to 70%). Moreover, the price of diode lasers is getting more and more competitive in relation to other systems. However, the significant drawback of this technology is the scarcity of diode lasers with short and high power pulses, important, e.g., in the case of selective photothermolises therapy [3]. Pulse powers up to 150 W are available [59].

## Figures and Tables

**Table 1:** Medical application of pulsed and cw diode laser with wavelengths 810 and 980 nm (λ denotes wavelengths, τ pulse lengths).

λ (nm)	Operation Mode	Applications Field	Ref.
810 ± 10	cw and pulsed	photobiomodulation (PBM)—also known as biostimulation or low level laser therapy—LLLT, dental biostimulation, neuronal differentiation	[4,48]
	τ ~ 5–15 ms ok. 30 Hz	lesion tissue: telangiectasias, spider veins, capillary dermal malformation and a cutaneous venous malformation	[10]
	NA	vascular ophtalmology,	[49]
	cw and pulsed	congenital subglottic hemangiomas	[13]
	cw	Tis and T1 glottic carcinomas	[14]
	cw, 3.5 W	Pyogenic granuloma	[16]
	8–10 W τ~ 0.5–1 s	Laser assisted dacryocystorhinostomy	[43]
	p. 30 ms, 7 Hz	hair removal	[21]
	5 W, τ = 4 s	Laryngology: laser-assisted somnoplasty, frenuloplasty, closure of tonsillar crypts, haemostasis, removal of cancerous changes, blepharoplasty, surgery of laryngopharynx and larynx, separation of adhesions in nasal septum	here
980 ± 10	cw, pulsed	photobiomodulation (PBM)—also known as biostimulation or low level laser therapy—LLLT, wound and ulcers healing applications	[7]
	pulsed, cw	tissue tumors, frenectomies, excision of gingival hyperplasias, vestibuloplasties, hemangioma removal, and periimplant	[11]
	NA	turbinate reduction, nasal polypectomy, ablation of an oral papilloma, photocoagulation of nasal telangiectasias,	[23]
	NA	endovenous laser treatment of saphenous veins	[24]
	NA	telangiectasias	[12]
	cw	early glottic cancer	[27]
	cw	vocal fold polyps	[28]
	Ceralas D15, 12 W superpulse	laryngeal lesions: vocal polyps, leucoplakia, laryngeal hair, granuloma, subglottic stenosis and tracheal lesions	[29]
	cw,	dermatology: neurofibroma, hemangioma	[34]
	τ = 50 ms, 10 Hz		
	dual 980/1470 nm	vascular lesions of skin and lips: cherry angiomas, venous lakes, lip hemangioma, and spider nevi, couperose, facial telangiectasia	[50]
	cw, 30–120 W	prostate treatment	[51]
	τ = 0.1 s, 5–9 Hz		
	NA	ophthalmology	[47]
	3 W (100 Hz)	removal of intracanal *Enterococcus faecalis* biofilm	[32]
	cw 8 to 9 W	bilateral vocal fold immobility (BVFI)	[30,31]
	cw 3–4 W	Maxillofacial surgeries including intrinsic TMJ pathologies	[45]
	cw 1.5 W	gingival depigmentation	[52]

## Appendix A. Patient Statement of Informed Consent for Surgical Treatment

As a patient of MML Medical Center, I hereby agree to undergo treatment, which will consist of:

(Name of procedure)

I have been informed of the requirements, processes and stages of surgical treatment, its purpose, expected results and potential risks that may occur as a result of this treatment. I accept the multi-disciplinary treatment plan, which involves surgical treatment. I have been informed of the costs of treatment and accept these.

I have been informed of the possibility of early and late post-surgery complications and the accompanying risks. I have understood the explanations and asked all questions that are of interest to me in regard to this medical procedure. Should a situation arise requiring it, I agree to a modification of the surgical procedure to the necessary extent, in accordance with the principles of medical knowledge.

I hereby give conscious consent to perform this treatment under local/general anaesthesia and declare that I have not concealed any crucial information regarding my overall health status. I have been informed about the possibilities of medical complications during the procedure which will be conducted.

I have been informed of and agree to allow the necessary photographic and radiological documentation in connection with the treatments.

I have been informed about the necessity of reporting to post-treatment follow-up control visits. I have been informed that smoking and poor oral hygiene and failure to follow post-treatment recommendations can significantly exacerbate potential post-treatment complications and negatively affect the treatments success.

I submit to the following restrictions associated with the medical procedure:

Performing Doctor               Legible Patient signature

## References

[1] Goldman L., Blaney D.J., Kindel D.J., Franke E.K. (1963). Effect of the Laser Beam on the Skin**From the Departments of Dermatology and Physics of the University of Cincinnati. The study was done under a grant from the U.S. Public Health Service 0H0018. J. Investig. Dermatol..

[2] Song K.U. (2017). Footprints in Laser Medicine and Surgery: Beginnings, Present, and Future. Med. Lasers.

[3] Anderson R., Parrish J. (1983). Selective photothermolysis: Precise microsurgery by selective absorption of pulsed radiation. Science.

[4] Mester A. (2013). Laser Biostimulation. Photomed. Laser Surg..

[5] Hamblin M.R. (2016). Photobiomodulation or low-level laser therapy. J. Biophotonics.

[6] Mester E., Szende B., Spiry T., Scher A. (1972). Stimulation of wound healing by laser rays. Acta Chir. Acad. Sci. Hung..

[7] Kawalec J., Reyes C., Penfield V., Hetherington V., Hays D., Feliciano F., Gartz D., Jones J., Esposito R., Cernica M. (2001). Evaluation of the Ceralas D15 diode laser as an adjunct tool for wound care: A pilot study. Foot.

[8] Goo H., Kim H., Ahn J.-C., Cho K.J. (2019). Effects of Low-level Light Therapy at 740 nm on Dry Eye Disease In Vivo. Med. Lasers.

[9] Espey B.T., Kielwein K., van der Ven H., Steger K., Allam J., Paradowska-Dogan A., van der Ven K. (2021). Effects of Pulsed-Wave Photobiomodulation Therapy on Human Spermatozoa. Lasers Surg. Med..

[10] Bass L.S. (1995). Photosclerosis of cutaneous vascular malformations with a pulsed 810-nm diode laser. Lasers in Surgery: Advanced Characterization, Therapeutics, and Systems V, Proceedings of the Photonics West '95, San Jose, CA, USA, 1−28 February 1995.

[11] Romanos G., Nentwig G.-H. (1999). Diode Laser (980 nm) in Oral and Maxillofacial Surgical Procedures: Clinical Observations Based on Clinical Applications. J. Clin. Laser Med. Surg..

[12] Desiate A., Cantore S., Tullo D., Profeta G., Grassi F.R., Ballini A. (2009). 980 nm diode lasers in oral and facial practice: Current state of the science and art. Int. J. Med. Sci..

[13] Saetti R., Silvestrini M., Cutrone C., Narne S. (2008). Treatment of Congenital Subglottic Hemangiomas. Arch. Otolaryngol. Head Neck Surg..

[14] Ferri E., Armato E. (2008). Diode laser microsurgery for treatment of Tis and T1 glottic carcinomas. Am. J. Otolaryngol..

[15] Mittnacht D., Linder A., Foth H.-J. (2005). Medical Application of a High Power Diode Laser. Therapeutic Laser Applications and Laser-Tissue Interactions II, In Proceedings of the SPIE—The International Society for Optical Engineering, Munich, Germany, 12–16 Jun 2005.

[16] Azma E., Safavi N. (2013). Diode Laser Application in Soft Tissue Oral Surgery. J. Lasers Med. Sci..

[17] Lee D.Y., Cho J.-G., Im N.-R., Lee H.-J., Kim B., Jung K.-Y., Kim T.H., Baek A.S.-K. (2015). Evaluation of the Efficacy of 1940-nm Diode Laser in Tonsillectomy: Preliminary Report. Med. Lasers.

[18] Kang S.H., Lim S., Oh D., Kang K., Jung K.J., Kim H.K., Lee S.H., Baek S.-K., Kim A.T.H. (2015). Clinical Feasibility Trial of 1,940-nm Diode Laser in Korean Patients with Inferior Turbinate Hypertrophy. Med. Lasers.

[19] Agrawal A.A., Prabhu R., Sankhe R., Wagle S.V. (2018). Influence of external chromophore on cutting efficacy of 940 nm diode laser: An In vitro animal tissue study. Contemp. Clin. Dent..

[20] Im N.-R., Kim B., Kim J., Baek S.-K. (2019). Treating Oral Leukoplakia with a 532-nm Pulsed Diode Laser. Med. Lasers.

[21] Kang H.Y., Park E.S., Nam S.M. (2018). Prospective, Comparative Evaluation of Forearm and Lower Leg Hair Removal with 808-nm Diode Laser at Different Fluences. Med. Lasers.

[22] Im N.-R., Moon J., Choi W., Kim B., Lee J.J., Kim H., Baek S.-K. (2018). Histologic Evaluation of Blood Vessels Sealed with 1,470-nm Diode Laser: Determination of Adequate Condition for Laser Vessel Sealing. Med. Lasers.

[23] Newman J., Anand V. (2002). Applications of the diode laser in otolaryngology. Ear Nose Throat J..

[24] Schmedt C.-G., Sroka R., Steckmeier S., Meissner O.A., Babaryka G., Hunger K., Ruppert V., Sadeghi-Azandaryani M., Steckmeier B.M. (2006). Investigation on Radiofrequency and Laser (980 nm) Effects after Endoluminal Treatment of Saphenous Vein Insufficiency in an Ex-vivo Model. Eur. J. Vasc. Endovasc. Surg..

[25] Reynaud J.P., Skibinski M., Wassmer B., Rochon P., Mordon S. (2009). Lipolysis using a 980-nm diode laser: A retrospective analysis of 534 procedures. Aesthetic Plast. Surg..

[26] Weiss R.A., Beasley K. (2009). Laser-assisted liposuction using a novel blend of lipid- and water-selective wavelengths. Lasers Surg. Med..

[27] Tunçel Ü., Cömert E. (2013). Preliminary Results of Diode Laser Surgery for Early Glottic Cancer. Otolaryngol. Neck Surg..

[28] Karasu M.F., Gundogdu R., Cagli S., Aydin M., Arli T., Aydemir S., Yüce I., Aydın M. (2014). Comparison of Effects on Voice of Diode Laser and Cold Knife Microlaryngology Techniques for Vocal Fold Polyps. J. Voice.

[29] Hwang S.M., Lee D.Y., Im N.-R., Lee H.-J., Kim B., Jung K.-Y., Kim T.H., Baek A.S.-K. (2015). Office-Based Laser Surgery for Benign Laryngeal Lesion. Med. Lasers.

[30] Karkos P.D., Stavrakas M. (2015). Minimizing revision rates with the “Π” technique for bilateral vocal fold immobility: A new technique combining carbon dioxide and diode laser. Head Neck.

[31] Karkos P.D., Stavrakas M., Koskinas I., Markou K., Triaridis S., Constantinidis J. (2021). 5 Years of Diode Laser “Π” Technique for Bilateral Vocal Fold Immobility: A Technique That Improves Airway and Is Friendly to the Voice. Ear Nose Throat J..

[32] Prażmo E., Godlewska A., Sałkiewicz M., Mielczarek A. (2017). Effects of 980 nm diode laser application protocols on the reduction of Enterococcus faecalis intracanal biofilm: An in vitro study. Dent. Med. Probl..

[33] Wróbel M.S., Jędrzejewska-Szczerska M., Galla S., Piechowski L., Sawczak M., Popov A.P., Bykov A.V., Tuchin V.V., Cenian A. (2015). Use of optical skin phantoms for preclinical evaluationof laser efficiency for skin lesion therapy. J. Biomed. Opt..

[34] Szymańczyk J., Sawczak M., Cenian W., Karpienko K., Jędrzejewska-Szczerska M., Cenian A. (2017). Application of the laser diode with central wavelength 975 nm for the therapy of neurofibroma and hemangiomas. J. Biomed. Opt..

[35] Milanic M., Cenian A., Verdel N., Cenian W., Stergar J., Majaron B. (2019). Temperature Depth Profiles Induced in Human Skin In Vivo Using Pulsed 975 nm Irradiation. Lasers Surg. Med..

[36] Piechowski L., Cenian W., Sawczak M., Cenian A. (2012). Pulsed dermatologic 20W diode-laser emitting at 975 nm. Laser Technology 2012: Applications of Lasers, Proceedings of the Tenth Symposium on Laser Technology, Bellingham, WA, USA, 24−28 September 2012.

[37] Bodnar T., Galdi G.P., Necasova S. (2014). Fluid-Structure Interaction and Biomedical Applications.

[38] Badur J., Ziółkowski P., Zakrzewski W., Sławiński D., Kornet S., Kowalczyk T., Hernet J., Piotrowski R., Felincjancik J., Ziółkowski P.J. (2014). An advanced Thermal–FSI approach to flow heating/cooling. J. Phys. Conf. Ser..

[39] Ziółkowski P.J., Ochrymiuk T., Eremeyev V.A. (2019). Adaptation of the arbitrary Lagrange–Euler approach to fluid–solid interaction on an example of high velocity flow over thin platelet. Contin. Mech. Thermodyn..

[40] Maitland D.J., Eder D.C., London R.A., Glinsky M.E., Soltz B.A. (1996). Dynamic simulations of tissue welding. Proc. Soc. Photo-Opt. Ins..

[41] Zhou J., Liu J., Yu A. (2005). Numerical Study on the Thawing Process of Biological Tissue Induced by Laser Irradiation. ASME. J. Biomech. Eng..

[42] Michalik M., Podbielska-Kubera A., Dmowska-Koroblewska A. (2018). Diode laser-assisted uvulopalatoplasty using palisade technique. New Med..

[43] Broda M., Michalik M., Białas D., Różycki R. (2018). Laser dacryocystorhinostomy—The lasers use in treatment of lacrimal duct obstruction. OphthaTherapy Ther. Ophthalmol..

[44] Michalik M. (2019). The influence of mitomycin C on clinical results of the nasolacrimal duct anastomosis using the diode laser. OphthaTherapy Ther. Ophthalmol..

[45] Guerra R.C., de Luca D.N., Pereira R.S., Carvalho P.H.A., Homsi N., Radaic P., Pastore G.P., Hochuli-Vieira E. (2021). TMJ diode surgical laser approach in a contemporary treatment of tempomandibular joint pathologies. A technical note. Oral Surg..

[46] Hanke A., Fimmers R., Frentzen M., Meister J. (2021). Quantitative determination of cut efficiency during soft tissue surgery using diode lasers in the wavelength range between 400 and 1500 nm. Lasers Med. Sci..

[47] Goel R., Nagpal S., Garg S., Malik K.P.S. (2015). Is transcanalicular laser dacryocystorhinostomy using low energy 810 nm diode laser better than 980 nm diode laser?. Oman J. Ophthalmol..

[48] George S., Hamblin M.R., Abrahamse H. (2020). Photobiomodulation-Induced Differentiation of Immortalized Adipose Stem Cells to Neuronal Cells. Lasers Surg. Med..

[49] Akduman L., Olk R.J. (1997). Diode Laser (810 nm) versus Argon Green (514 nm) Modified Grid Photocoagulati-on for Diffuse Diabetic Macular Edema. Ophthalmology.

[50] Wollina U., Goldman A. (2020). The dual 980-nm and 1470-nm diode laser for vascular lesions. Dermatol. Ther..

[51] Wendt-Nordahl G., Huckele S., Honeck P., Alken P., Knoll T., Michel M.S., Häcker A. (2007). 980-nm Diode Laser: A Novel Laser Technology for Vaporization of the Prostate. Eur. Urol..

[52] Arif R.H., Kareem F.A., Zardawi F.M., Al-Karadaghi T.S. (2021). Efficacy of 980 nm diode laser and 2940 nm Er: YAG laser in gingival depigmentation: A comparative study. J. Cosmet. Dermatol..

[53] Betka J., Plzák J., Zábrodský M., Kastner J., Boucek J. (2013). Lasers in otorhinolaryngology (ORL) and head and neck surgery. Lasers for Medical Applications.

[54] Abiri A., Bs K.G., Maducdoc M., Sahyouni R., Wang M.B., Kuan E.C. (2020). Laser-Assisted Control of Epistaxis in Hereditary Hemorrhagic Telangiectasia: A Systematic Review. Lasers Surg. Med..

[55] Ferlito S., Nane S., Grillo C., Maugeri M., Cocuzza S., Grillo C. (2011). Diodes laser in ENT surgery. Acta Med. Mediterr..

[56] Massaro B.M., Gonnering R.S., Harris G.J. (1990). Endonasal laser dacryocystorhinostomy. A new approach to nasolacrimal duct obstruction. Arch. Ophthalmol..

[57] Silkiss R.Z., Axelrod R.N., Iwach A.G., Vassiliadis A., Hennings D.R. (1992). Transcanalicular THC: YAG dacryocystorhinostomy. Ophthalmic Surg..

[58] Nowak R., Rekas M., Gospodarowicz I.N., Ali M.J. (2021). Long-term outcomes of primary transcanalicular laser dacryocystorhinostomy. Graefe’s Arch. Clin. Exp. Ophthalmol..

[59] Yaroslavsky I., Boutoussov D., Vybornov A., Perchuk I., Meleshkevich V., Altshuler G.B. (2018). Ex vivo evaluation of super pulse diode laser system with smart temperature feedback for contact soft-tissue surgery. Lasers in Dentistry XXIV, Proceedings of the SPIE BiOS, Bellingham, WA, USA, 27 January−1 February 2018.

